# MEOX2: a homeobox transcription factor with multifaceted biological functions and broad disease associations

**DOI:** 10.3389/fcell.2026.1895187

**Published:** 2026-07-23

**Authors:** Yi Liu, Minming Yi, Chunrong Tang, Taiqiang Wang, Xuan Li

**Affiliations:** 1 West China Hospital Sichuan University Jintang Hospital, Jintang First People’s Hospital, Chengdu, China; 2 Sichuan Jiaotong Hospital, Chengdu, China

**Keywords:** biomarker, MEOX2, therapeutic target, transcription factor, tumour microenvironment

## Abstract

The mesenchymal homeobox transcription factor MEOX2 is recognized for its roles in development and homeostasis, but the full landscape of its complex and often contradictory functions in human diseases, along with its potential unifying regulatory logic, remains to be systematically elucidated. This Review synthesizes current evidence to provide a comprehensive overview of MEOX2’s widespread roles in both neoplastic and non-neoplastic diseases. MEOX2 is remarkably context-dependent: it acts as an oncogene in glioblastoma and lung cancer, but as a tumour suppressor in breast cancer, hepatocellular carcinoma and other tumour types. Its dysregulation is also linked to neurovascular deficits in Alzheimer disease (AD), cardiovascular disorders, metabolic fibrosis and developmental malformations. This functional versatility stems from MEOX2’s role as a key signalling integrator, subject to fine-tuned regulation by epigenetic mechanisms, non-coding RNA networks and core pathways including PI3K/AKT, ERK and Hedgehog (Hh). Given the strong association between MEOX2 expression and clinical outcomes, it has emerged as a potential diagnostic and prognostic biomarker for several diseases. Intervention strategies targeting MEOX2 and its regulatory networks show translational promise. This Review proposes a conceptual framework placing MEOX2 as a ‘cross-disease core regulatory node’, systematically delineates its complex disease associations and molecular mechanisms, and charts a course for the future development of precision medicine strategies targeting MEOX2.

## 1 Introduction

Transcription factors encoded by the homeobox gene family act as foundational determinants of pattern formation and cell fate during embryonic development. MEOX2, a member of this family, has seen its biological roles continually expanded and redefined since its discovery (Grigoriou et al., 1995). Early studies focused on its antiproliferative effects in vascular smooth muscle and endothelial cells, as well as its expression patterns during cardiovascular development (Skopick et al., 1997; Gorski et al., 1993). However, with the advent of high-throughput sequencing and bioinformatics, aberrant MEOX2 expression patterns in human diseases, particularly cancer, have been extensively uncovered.

MEOX2 is highly context-dependent, making it an intriguing yet challenging target. In GBM, it is upregulated and linked to poor survival (Schönrock et al., 2022; Tachon et al., 2019); in BC, its downregulation predicts worse outcomes (Wang et al., 2022a; Wang et al., 2022b). This ‘dual’ role is also reflected in its regulatory mechanisms: MEOX2 is not only regulated by epigenetic modifications (e.g., DNA methylation, histone modifications) but is also a direct target of numerous microRNAs (miRNAs) and long non-coding RNAs (lncRNAs), forming an intricate gene regulatory network (Tachon et al., 2021; Liu et al., 2025; Liu X.-X. et al., 2021). Beyond cancer, the roles of MEOX2 in neurodegenerative diseases, cardiovascular pathologies, fibrotic disorders and congenital malformations are gaining increasing attention. In AD, loss of MEOX2 expression in brain endothelial cells is considered a key contributor to blood–brain barrier dysfunction and impaired β-amyloid clearance (Wu et al., 2005). In the heart, it participates in fibrotic responses and heart failure (HF) progression (Cunnington et al., 2014; Gao et al., 2025). During development, MEOX2 is a critical regulator of anteroposterior patterning of the palate, and its dysfunction can lead to cleft palate (Jin and Ding, 2006; Li and Ding, 2007).

This Review aims to systematically assess the biological characteristics of MEOX2 and comprehensively synthesize its expression patterns, molecular mechanisms and clinical significance across a spectrum of human diseases, including both neoplastic and non-neoplastic conditions. We further discuss the translational potential of MEOX2 as a diagnostic biomarker and therapeutic target, and offer perspectives on future research directions in light of current limitations. Crucially, MEOX2 plays seemingly contradictory roles in different diseases–acting as a *bona fide* oncogene in GBM and lung cancer, while often functioning as a tumour suppressor in BC and HCC. Simultaneously, MEOX2 deficiency is a key driver of vascular dysfunction in AD, while it may serve as a protective factor in liver fibrosis. This extreme context-dependency makes MEOX2 a particularly challenging yet promising research target. This Review provides an updated and comprehensive synthesis of MEOX2’s roles across neoplastic and non-neoplastic diseases, establishing it as a cross-disease core regulatory node. By delineating its complex regulatory networks, bidirectional pathological functions and broad translational potential, this Review not only offers a comprehensive knowledge map but also aspires to provide an innovative theoretical framework for the future development of interdisciplinary precision medicine strategies predicated on MEOX2 status.

## 2 Molecular biological characteristics of MEOX2

### 2.1 Gene, protein structure and evolutionary conservation

The MEOX2 gene is located on human chromosome 7p22.1-p21.3 (Grigoriou et al., 1995). The encoded protein contains several characteristic domains: an N-terminal domain, a histidine/glutamine-rich (His/Gln-rich) region and a C-terminal homeodomain (Candia et al., 1993). The homeodomain is the core region responsible for binding to specific DNA sequences and exerting transcriptional regulatory functions. Studies have shown that the middle region of the MEOX2 protein (between the His/Gln-rich region and the homeodomain, amino acids 101–185) is a critical region for interaction with partner proteins such as RING finger protein 10 (RNF10) (Lin et al., 2005). MEOX2 is highly evolutionarily conserved, with its homeodomain being completely identical in humans, rodents and even amphibians, suggesting it performs essential biological functions in vertebrates (Grigoriou et al., 1995).

**FIGURE 1:**
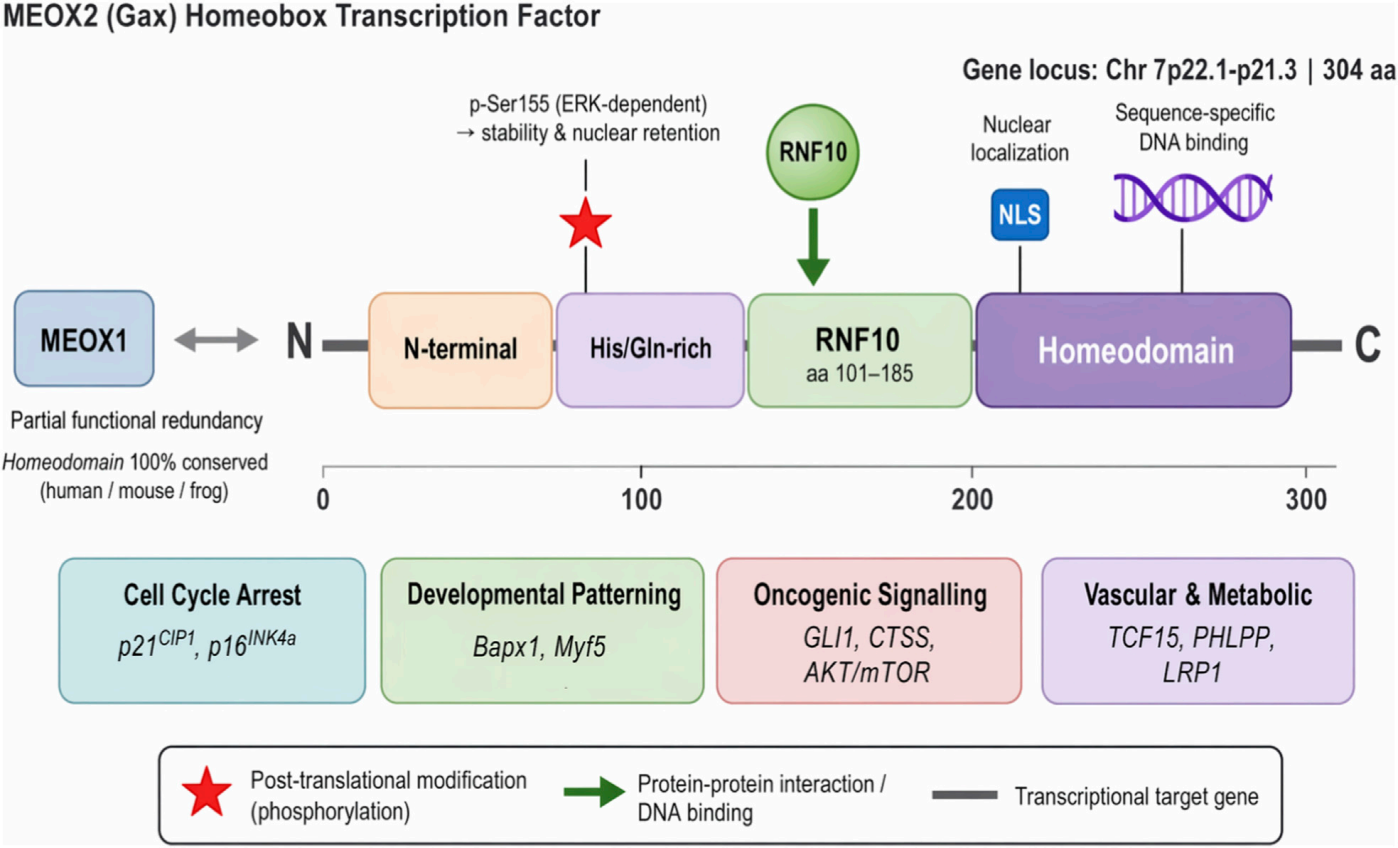
Domain architecture and functional versatility of MEOX2. Schematic representation of the MEOX2 protein (304 amino acids) encoded by the gene located on chromosome 7p22.1–p21.3. MEOX2 contains an N-terminal domain, a histidine/glutamine-rich (His/Gln-rich) region, a central RNF10-binding domain (amino acids 101–185), and a C-terminal homeodomain that harbours a nuclear localization signal (NLS) and mediates sequence-specific DNA binding. MEOX1 exhibits partial functional redundancy with MEOX2, and their homeodomains are 100% conserved across human, mouse, and frog. Post-translational phosphorylation at Ser155 by ERK stabilizes the protein and promotes nuclear retention. The lower panel summarizes the context-dependent functional outputs of MEOX2: induction of G0/G1 cell cycle arrest via activation of p21CIP1 and p16INK4a; regulation of developmental patterning through direct activation of Bapx1 (sclerotome differentiation) and Myf5 (limb myogenesis); promotion of oncogenic signalling via transcriptional activation of GLI1 and CTSS and context-dependent modulation of AKT/mTOR (activated in glioblastoma and lung cancer, inhibited in breast cancer); and vascular and metabolic regulation through TCF15-mediated cardiac fatty acid uptake, PHLPP-dependent inhibition of hepatic stellate cell proliferation, and LRP1-mediated amyloid-β clearance at the blood–brain barrier.

MEOX2 and MEOX1 exhibit partial functional redundancy during development but have distinct expression patterns (Reijntjes et al., 2007). Both are homeodomain-containing transcription factors that share a highly conserved DNA-binding homeodomain (Grigoriou et al., 1995). MEOX1 is evolutionarily conserved across vertebrates, and its protein has been shown to occupy conserved promoter regions of target genes such as Tbx18 and Uncx (Skuntz et al., 2009). The “partial functional redundancy” between MEOX1 and MEOX2 refers to the observation from mouse knockout studies that single-gene deletions produce relatively mild, tissue-restricted phenotypes—MEOX1 deficiency primarily affects sclerotome-derived axial structures (Skuntz et al., 2009), whereas MEOX2 loss impacts limb muscle development (Otto et al., 2010)—whereas double knockouts result in severe synthetic phenotypes, including complete loss of axial skeleton and severe skeletal muscle deficiency (Mankoo et al., 2003). This indicates that the two genes share overlapping functions in certain developmental contexts but also execute non-redundant roles in others. Mechanistically, both MEOX1 and MEOX2 can activate common target genes such as p21CIP1/WAF1 and p16INK4a, though through distinct transcriptional mechanisms (Douville et al., 2011). The domain architecture and principal functional outputs of MEOX2 are summarized in Figure 1.

### 2.2 Expression and transcriptional regulation

MEOX2 expression is subject to multi-layered and finely tuned regulation. At the transcriptional level, its core promoter activity depends on multiple positive transcription factors, including Sp1, HRF-1 and members of the myocyte enhancer factor 2 (MEF2/RSRF) family (Andrés et al., 1995). Mutation of the MEF2 site has a more pronounced effect in cells with high endogenous MEF2 activity, and overexpression of MEF2A transactivates the MEOX2 promoter, revealing a direct regulatory link between MADS-box transcription factors and homeobox genes.

Epigenetic modifications represent another crucial mechanism regulating MEOX2 expression. In lung cancer and glioma, MEOX2 overexpression is often accompanied by decreased repressive histone mark H3K27me3 and increased active mark H3K4me3 at its promoter region (Ávila-Moren et al., 2014; Armas-López et al., 2017). MEOX2 promoter methylation influences its expression; in glioma stem cells, expression correlates with hypomethylation (Tachon et al., 2021). During lung development and in lung cancer, methylation of the 5′-UTR region of the MEOX2 gene is inversely correlated with gene expression (Cortese et al., 2008). In psoriasis, MEOX2 has also been identified as one of the differentially expressed genes associated with N^6^-methyladenosine (m^6^A) methylation (Gan et al., 2024).

At the post-transcriptional level, MEOX2 is a target of multiple miRNAs, constituting an extensive regulatory network. The miR-130a and miR-301a families have been shown to directly target the 3′untranslated region (3′UTR) of MEOX2, suppressing its expression and playing roles in angiogenesis and HCC progression (Chen and Gorski, 2008; Permuth-Wey et al., 2015; Zhou et al., 2012a). Other miRNAs regulating MEOX2 include miR-148a-3p (in skeletal muscle satellite cell differentiation) (Yin et al., 2020), miR-143-3p (in cervical cancer (CC) indirectly via MEOX2-AS1) (Liu X.-X. et al., 2021) and miR-1200 (in gestational diabetes mellitus (GDM)) (Huang et al., 2021). Recently, circular RNAs (circRNAs) such as circ_0000190 have been found to act as competitive endogenous RNAs (ceRNAs) sponging miR-301a, thereby upregulating MEOX2 and inhibiting triple-negative BC (TNBC) progression (Liu et al., 2025). Additionally, circ_0074673 regulates endothelial cell function via the miR-1200/MEOX2 axis (Huang et al., 2021), and circINSR inhibits sheep adipogenesis differentiation via the miR-152/MEOX2 axis (Zhao et al., 2023). Long non-coding RNAs are also involved: MEOX2-AS1 acts as a ceRNA in CC (Liu X.-X. et al., 2021), LINC01936 potentially interacts with MEOX2 in lung squamous cell carcinoma (Tian et al., 2023) and NRAV is regulated by MEOX2 in gastric cancer (GC) (Beck-Cormier et al., 2014). Furthermore, miR-1 and miR-206 target Meox2 during C2C12 myoblast differentiation (Goljanek-Whysall et al., 2012). During induced pluripotent stem (iPS) cell generation, the miR-130/301/721 family enhances reprogramming efficiency by suppressing Meox2 (Pfaff et al., 2011).

### 2.3 Physiological functions in normal tissues

**FIGURE 2:**
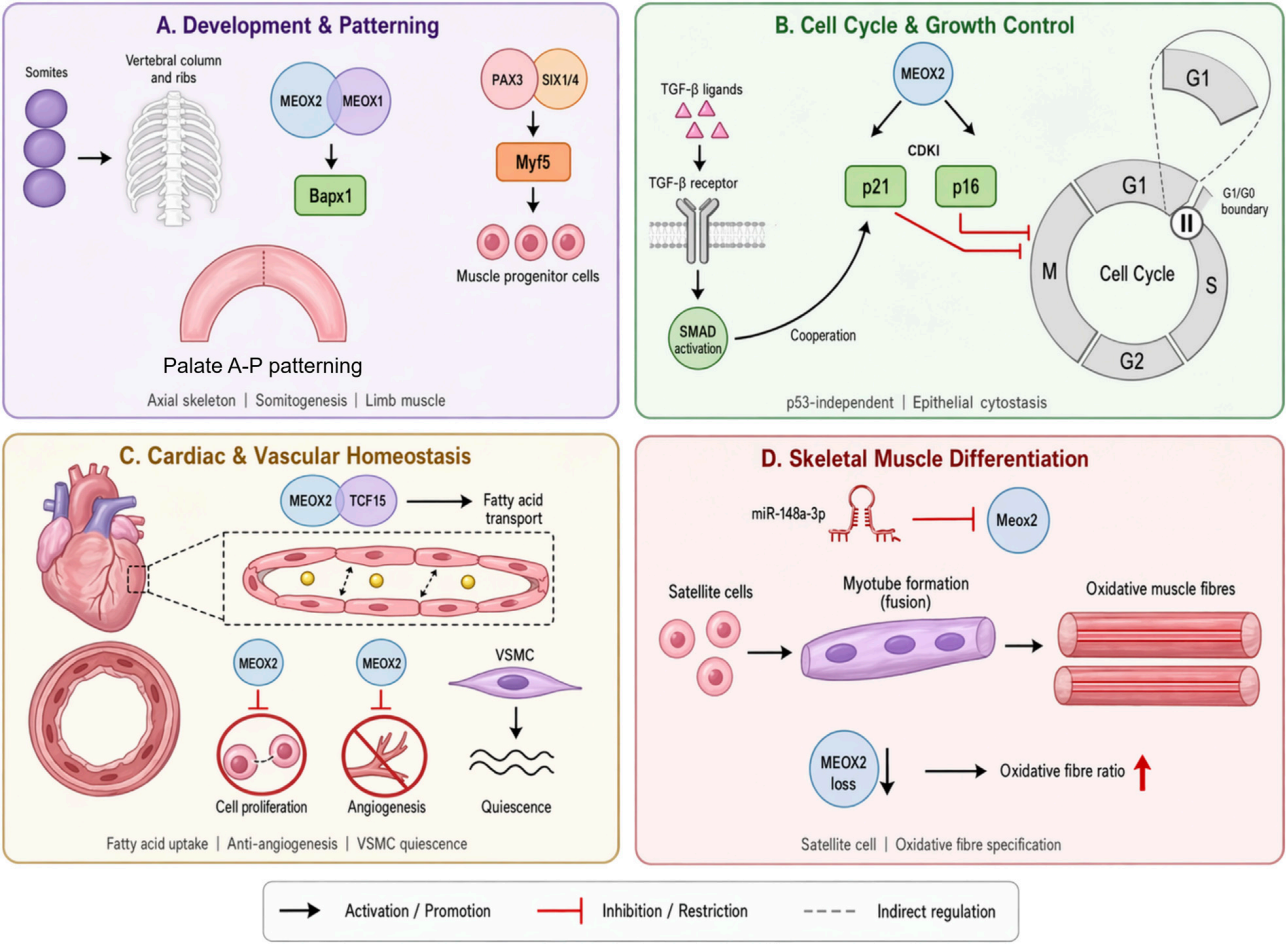
Physiological functions of MEOX2 in normal tissues. **(A)** Development and patterning: MEOX2 cooperates with MEOX1 to activate Bapx1 for sclerotome differentiation and axial skeleton formation, and interacts with PAX3/SIX1/4 to regulate Myf5 expression during limb myogenesis; MEOX2 expression defines the posterior palate and establishes anterior-posterior patterning. **(B)** Cell cycle and growth control: MEOX2 induces G0/G1 arrest via p53-independent activation of the cyclin-dependent kinase inhibitors p21CIP1 and p16INK4a, and cooperates with TGF-β/SMAD signalling to mediate epithelial cytostasis. **(C)** Cardiac and vascular homeostasis: the MEOX2/TCF15 heterodimer programs heart capillary endothelium for fatty acid transport; MEOX2 inhibits endothelial cell proliferation and angiogenesis, and maintains vascular smooth muscle cell quiescence. **(D)** Skeletal muscle differentiation: miR-148a-3p post-transcriptionally represses MEOX2; MEOX2 is required for myotube formation, and its loss leads to a compensatory switch toward oxidative muscle fibres.

Under physiological conditions, MEOX2 plays essential roles in embryonic development and tissue homeostasis. During cardiogenesis, MEOX2 exhibits a biphasic expression pattern: it is prominently expressed in the early cardiac tube and maintains expression through the looping stage, with a second wave of expression in the ventricular compact layer at later stages (Skopick et al., 1997). In the adult heart, MEOX2 forms a heterodimer with TCF15 to program the capillary endothelium for fatty acid uptake, thereby contributing to cardiac energy metabolism (Coppiello et al., 2015). In skeletal muscle development, Meox2 is required for proper myoblast proliferation and fibre-type specification; its loss leads to reduced foetal myoblast numbers and an increase in oxidative myofibres (Otto et al., 2010). MEOX2 also collaborates with MEOX1 upstream of somite formation and sclerotome differentiation (Mankoo et al., 2003; Rodrigo et al., 2004). In the vascular system, MEOX2 functions as a negative regulator of angiogenesis, suppressing endothelial cell proliferation and tube formation in response to pro-angiogenic stimuli (Gorski and Leal, 2003; Patel et al., 2005). These physiological roles establish the baseline from which MEOX2 dysregulation—whether through loss or gain of function—can drive distinct pathological outcomes in a tissue- and context-dependent manner. These context-dependent physiological roles of MEOX2 in embryonic development, cell cycle regulation, cardiovascular homeostasis and skeletal muscle differentiation are illustrated in Figure 2.

The observation that MEOX2 exerts opposing functions across different tumour types may be partially explained by this developmental and tissue-specific regulatory logic. As a transcription factor, MEOX2 does not operate in isolation; its transcriptional output is shaped by the availability of context-specific co-factors, epigenetic landscapes and signalling crosstalk that vary markedly between tissues of origin. For instance, in GBM and lung cancer, MEOX2 potentiates oncogenic signalling through the ERK and GLI1 axes (Schönrock et al., 2022; Armas-López et al., 2017; Peralta-Arrieta et al., 2022), whereas in breast and liver cancer, it predominantly suppresses tumour progression via inhibition of PI3K/AKT signalling (Cai et al., 2022; Zhou et al., 2012b). This context dependency is further compounded by the presence of tissue-specific post-translational modifications—such as ERK-dependent phosphorylation at Ser155 (Schönrock et al., 2022)—and differential engagement of non-coding RNA regulatory networks. Thus, developmental lineage and tissue origin provide a framework for understanding MEOX2’s duality, but the final outcome likely reflects a convergence of cell-intrinsic programmes, microenvironmental cues and genetic/epigenetic alterations. A more definitive mechanistic understanding will require systematic comparative analyses of the MEOX2 cistrome and interactome across different cellular contexts.

### 2.4 Molecular functions and downstream pathways

As a transcription factor, MEOX2 exerts its functions by regulating the expression of downstream target genes. A classic function is the induction of cell cycle arrest. MEOX2 directly binds to and activates the promoters of the cyclin-dependent kinase inhibitors p21 (CIP1/WAF1) and p16 (INK4a), thereby arresting cells in the G0/G1 phase (Douville et al., 2011; Irelan et al., 2009; Smith et al., 1997). This function is p53-independent and represents a core mechanism by which MEOX2 inhibits cell proliferation. MEOX2 also participates in TGF-β-induced epithelial cell growth inhibition via transcriptional activation of p21 (Valcourt et al., 2007).

Mechanistically, MEOX2 cooperates with SMAD proteins to form transcriptional complexes that synergistically activate p21 expression, thereby enhancing the cytostatic response to TGF-β stimulation (Valcourt et al., 2007). This interaction is mediated through the SMAD-binding sites in the p21 promoter, establishing MEOX2 as a direct transcriptional co-regulator of TGF-β/SMAD signalling (Valcourt et al., 2007). Importantly, MEOX2 also exerts TGF-β-independent regulation of the cell cycle, as demonstrated by its direct activation of p21 and p16 promoters through homeodomain-mediated DNA binding in the absence of TGF-β stimulation (Douville et al., 2011; Smith et al., 1997; Petrovic et al., 2010). This p53-independent mechanism operates in multiple cell types, indicating that MEOX2 acts both downstream of TGF-β and as an autonomous cell cycle regulator (Douville et al., 2011; Smith et al., 1997; Petrovic et al., 2010).

MEOX2 acts as an integration node and modulator of several key signalling pathways. In glioma and lung cancer, MEOX2 enhances signalling through the ERK/MAPK and PI3K/AKT/mTOR pathways (Schönrock et al., 2022; Tachon et al., 2021; Cai et al., 2022). In lung cancer, MEOX2 forms a regulatory axis with GLI1, a key effector of the Hh pathway. MEOX2 directly occupies the promoter region of the GLI1 gene and activates its transcription, thereby promoting tumour progression and therapy resistance (Armas-López et al., 2017). Furthermore, MEOX2 has been shown to interact with the NF-κB pathway components p65 and IκBβ in the nucleus, exerting bidirectional modulation of NF-κB activity depending on its expression level, thereby influencing inflammatory and angiogenic responses (Chen et al., 2010). MEOX2 also participates in liver fibrosis and laryngeal cancer (LC) progression by regulating PI3K/AKT signalling (Wang et al., 2024; Tian et al., 2018). In cardiomyocytes, MEOX2 regulates AP-1 to participate in chemotherapy-induced cardiotoxicity (Shi et al., 2023; Duan et al., 2023).

During development, MEOX2 cooperates with MEOX1 and is essential for somite formation, patterning and differentiation (Mankoo et al., 2003). MEOX2 directly activates the transcription of Bapx1, a key gene for sclerotome differentiation, acting as an upstream regulator of axial skeletal development (Rodrigo et al., 2004). In limb muscle development, MEOX2 cooperates with factors such as PAX3 and SIX1/4 to finely control the temporal expression of the myogenic determination gene Myf5 (Buchberger et al., 2007; Daubas et al., 2015). In heart development, the MEOX2/TCF15 heterodimer regulates fatty acid uptake by heart capillary endothelial cells (Coppiello et al., 2015). In summary, MEOX2, as a transcription factor, directly induces G0/G1 cell cycle arrest by activating p21 and p16, and acts as an integration node for several critical signalling pathways (ERK/MAPK, PI3K/AKT/mTOR, Hh, NF-κB, AP-1), regulating tumour progression, therapy resistance, fibrosis and developmental processes.

## 3 Expression and function of MEOX2 in neoplastic diseases

**FIGURE 3:**
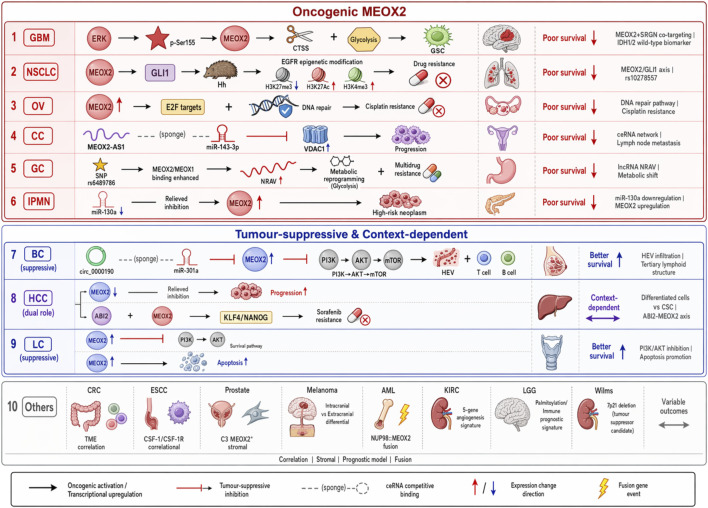
Context-dependent roles of MEOX2 in human neoplastic diseases. The left panel summarizes oncogenic MEOX2 in glioblastoma (GBM), where ERK-dependent phosphorylation at Ser155 stabilizes MEOX2, which activates CTSS and glycolysis to maintain glioma stem cell (GSC) stemness; in non-small cell lung cancer (NSCLC), MEOX2 activates GLI1 and epigenetically modifies EGFR to confer drug resistance; in ovarian cancer (OV), MEOX2 upregulation activates E2F targets and DNA repair to mediate cisplatin resistance; in cervical cancer (CC), lncRNA MEOX2-AS1 sponges miR-143-3p to upregulate VDAC1; in gastric cancer (GC), SNP rs6489786 enhances MEOX2 binding to upregulate lncRNA NRAV, driving glycolysis and multidrug resistance; in intraductal papillary mucinous neoplasm (IPMN), miR-130a downregulation relieves MEOX2 inhibition. The middle panel shows tumour-suppressive and context-dependent MEOX2 in breast cancer (BC), where circ_0000190 sponges miR-301a to upregulate MEOX2, which inhibits PI3K-AKT-mTOR and promotes high endothelial venule (HEV) infiltration; in hepatocellular carcinoma (HCC), MEOX2 loss promotes progression in differentiated cells, whereas ABI2-recruited MEOX2 activates KLF4/NANOG in cancer stem cells (CSCs) to confer sorafenib resistance; in laryngeal cancer (LC), MEOX2 upregulation inhibits PI3K-AKT and promotes apoptosis. The right panel illustrates other malignancies with correlative, stromal, prognostic or fusion-based MEOX2 associations: colorectal cancer (CRC), oesophageal squamous cell carcinoma (ESCC), prostate cancer, melanoma, acute myeloid leukaemia (AML), kidney renal clear cell carcinoma (KIRC), low-grade glioma (LGG), and Wilms tumour. Red arrows indicate oncogenic activation or poor-survival-associated upregulation; blue arrows indicate tumour-suppressive inhibition or better-survival-associated upregulation; double-headed arrows indicate context-dependent outcomes.

The role of MEOX2 in tumorigenesis and progression exhibits marked tissue specificity and disease context dependency, acting either as an oncogene or a tumour suppressor. To systematically synthesize the complex and context-dependent functions of MEOX2 across various tumour types, this section summarises the disease types, molecular mechanisms, signalling pathways and clinical associations in Table 1. The remarkably context-dependent functions of MEOX2 across diverse human malignancies—acting as an oncogene in some tumour types while functioning as a tumour suppressor in others—are systematically summarized in Figure 3.

**TABLE 1:** Mechanisms, molecular pathways and clinical associations of MEOX2 in various neoplastic diseases.

Disease category	Disease name	Mechanism of action	Molecular pathways	Clinical association	Ref.
Central nervous system tumours	Glioblastoma (GBM)	Oncogene: promotes proliferation, survival, sphere formation, inhibits apoptosis; potentiates ERK signalling (Ser155 phosphorylation maintains protein stability); transcriptionally activates CTSS; regulates metabolic reprogramming (glycolysis, hypoxia response); maintains GSC stemness and adhesion phenotype	ERK/MAPK, PI3K/AKT, hypoxia response/glycolysis pathways	High expression associated with shortened OS; biomarker for IDH1/2 wild-type subtype; aids pathological diagnosis (combined with SOX11, etc.); co-targeting MEOX2 and SRGN shows synergistic anti-tumour effect	Schönrock et al. (2022), Tachon et al. (2019), Tachon et al. (2021), Wang et al. (2022c), Proserpio et al. (2022), Lu et al. (2025), Soukup et al. (2023)
​	Low-grade glioma (LGG)	Prognosis-related gene; involved in palmitoylation- and immune microenvironment-related gene signatures	Palmitoylation; immune infiltration	Included in multi-gene prognostic models (e.g., CHI3L1, IGFBP2, MEOX2) for risk stratification	Wu et al. (2025), Wang et al. (2025a), Du et al. (2024)
Digestive system tumours	Hepatocellular carcinoma (HCC)	Mainly tumour suppressor: low expression promotes progression; but in liver CSCs, ABI2 recruits MEOX2 to activate KLF4/NANOG, maintaining stemness and promoting sorafenib resistance	PI3K/AKT (tumour suppressor in fibrosis, but different in CSCs); KLF4/NANOG axis	Low expression correlates with high Edmondson grade, vascular invasion, capsule invasion and short survival; ABI2-MEOX2 axis is a potential therapeutic target	Zhou et al. (2012b), Chen et al. (2022)
​	Oesophageal squamous cell carcinoma (ESCC)	Associated with tumour-associated macrophage infiltration	Possibly via CSF-1/CSF-1R signalling axis	Serves as an immune-related biomarker	Wang et al. (2021)
​	Colorectal cancer (CRC)	Tumour microenvironment-related prognostic gene	Correlates with immune cell infiltration levels	Included as one of six-gene prognostic models	Liu et al. (2021b)
​	Gastric cancer (GC)	Functional SNP rs6489786 enhances MEOX2 binding, upregulating oncogenic lncRNA NRAV; involved in multidrug resistance	Glucose metabolic reprogramming (shift from aerobic respiration to glycolysis)	Associated with GC development and drug resistance	Beck-Cormier et al. (2014), Yan et al. (2014)
​	Pancreatic intraductal papillary mucinous neoplasm (IPMN)	miR-130a downregulation leads to MEOX2 upregulation	miRNA-mRNA regulation	Associated with high-risk IPMN	Permuth-Wey et al. (2015)
​	Wilms tumour (nephroblastoma)	Located within homozygous deletion region at 7p21; candidate tumour suppressor	Possibly involved in angiogenesis	Potential tumour suppressor	Ohshima et al. (2009), Blish et al. (2010)
Thoracic tumours	Non-small cell lung cancer (NSCLC)	Oncogene: promotes chemotherapy resistance (cisplatin, EGFR-TKIs); activates GLI1 transcription; epigenetically regulates EGFR (reduces H3K27me3, increases H3K27Ac/H3K4me3)	MEOX2/GLI1 axis, EGFR/AKT/ERK, Hh pathway	High expression associated with poor prognosis and chemotherapy resistance; MEOX2/GLI1 axis is a target to overcome resistance; genetic variant rs10278557 correlates with clinical stage	Ávila-Moren et al. (2014), Armas-López et al. (2017), Tian et al. (2023), Peralta-Arrieta et al. (2022), Trejo-Villegas et al. (2025), Frullanti et al. (2011), Sawada et al. (2022)
​	Breast cancer (BC)	Tumour suppressor: low expression correlates with poor survival; overexpression inhibits proliferation, metastasis, enhances chemosensitivity; circ_0000190/miR-301a axis upregulates MEOX2; associated with tumour-associated HEVs and T/B-cell infiltration	PI3K/AKT/mTOR, ERK1/2, oxidative stress-RGS5	Low expression predicts poor prognosis; diagnostic and prognostic biomarker; angiogenesis-related; high expression in HEVs suggests better survival and immunotherapy response	Wang et al. (2022a), Wang et al. (2022b), Liu et al. (2025), Cai et al. (2022), Sawada et al. (2022), Tang et al. (2025), Liu and Wang (2021)
Reproductive system tumours	Ovarian cancer (OV)	High expression mediates cisplatin resistance; silencing MEOX2 enhances cisplatin sensitivity	E2F targets, DNA repair pathways	High expression associated with poor prognosis and cisplatin resistance	Wang et al. (2025b)
​	Cervical cancer (CC)	lncRNA MEOX2-AS1 sponges miR-143-3p to upregulate VDAC1, promoting progression; hub gene for lymph node metastasis	ceRNA network (MEOX2-AS1/miR-143-3p/VDAC1)	High expression predicts worse clinical outcome; potential therapeutic target	Liu et al. (2021a), Ding et al. (2023)
Other tumours	Laryngeal cancer (LC)	Tumour suppressor: overexpression promotes apoptosis	PI3K/AKT pathway (inhibition)	Low expression correlates with progression; candidate therapeutic gene	Tian et al. (2018)
​	Acute myeloid leukaemia (AML)	Presence of NUP98:MEOX2 fusion gene	Fusion gene drives leukaemogenesis	Novel fusion partner associated with high risk	Chonabayashi et al. (2026)
​	Prostate cancer	Expressed in a CAF subpopulation (C3 MEOX2^+^)	Tumour stromal heterogeneity	May affect prognosis and immunotherapy	Ding et al. (2025)
​	Melanoma	Differential expression between intracranial and extracranial metastases; associated with disease progression	Downregulation of immune-related pathways; brain-like phenotype gene program	Potential marker gene for distinguishing intracranial metastases	Cardinale et al. (2022), Kraft et al. (2024)
​	Kidney renal clear cell carcinoma (KIRC)	Included as one of five-gene angiogenesis signature	Angiogenesis-related pathways	Prognostic prediction model (MEOX2, PLG, PROX1, TEK, TIMP1)	Zhou et al. (2025)

### 3.1 Central nervous system tumours

GBM is a paradigm for the oncogenic role of MEOX2. MEOX2 is significantly overexpressed in GBM tissues and GSCs, correlating with shortened overall survival (Schönrock et al., 2022; Tachon et al., 2019; Proserpio et al., 2022). Functionally, MEOX2 potentiates ERK signalling through a feed-forward mechanism in which ERK-dependent phosphorylation of MEOX2 at Ser155 is crucial for maintaining its protein level and nuclear localisation (Schönrock et al., 2022). Knockdown or knockout of MEOX2 effectively inhibits GSC proliferation and sphere-forming ability and induces apoptosis (Proserpio et al., 2022). Mechanistically, MEOX2 promotes glioma cell proliferation and motility by transcriptionally activating cathepsin S (CTSS) (Wang J. et al., 2022). Moreover, MEOX2 is involved in regulating metabolic reprogramming in GSCs; its knockdown leads to downregulation of genes involved in the hypoxic response and glycolysis, pathways intimately associated with therapy resistance in high-grade glioma (Proserpio et al., 2022). MEOX2 also contributes to GSC survival and adhesion phenotypes via the ERK/MAPK and PI3K/AKT pathways (Tachon et al., 2021). Importantly, MEOX2 expression correlates with the IDH1/2 wild-type molecular subtype, making it an interesting biomarker for this subtype of GBM (Tachon et al., 2019). Therapeutic strategies targeting MEOX2 are being explored; for instance, co-targeting MEOX2 (enriched in the proneural GSC state) and SRGN (enriched in the mesenchymal GSC state) shows synergistic anti-tumour effects in preclinical models (Lu et al., 2025). For diagnosis, immunohistochemical (IHC) detection of MEOX2, together with SOX11, INSM1 and EGFR, has been shown to help distinguish GBM cells from reactive gliosis in limited tissue samples (Soukup et al., 2023). In low-grade glioma (LGG), MEOX2 has been incorporated into prognostic models based on the tumour microenvironment or palmitoylation-related genes, demonstrating good risk stratification ability (Wu et al., 2025; Wang Z. et al., 2025; Du et al., 2024). Additionally, galectin-1 (Gal-1) expression correlates with MEOX2 in GBM (Martínez-Bosch et al., 2023). These findings establish MEOX2 as an oncogene and prognostic biomarker in GBM, with functions spanning proliferation, metabolic reprogramming and stemness maintenance. Its utility in prognostic models and potential as a therapeutic target underscore its translational value.

### 3.2 Digestive system tumours

In digestive system malignancies, the role of MEOX2 is predominantly tumour-suppressive in most epithelial-derived cancers, with a notable exception in liver cancer stem cells where it contributes to stemness maintenance and therapy resistance.

In hepatocellular carcinoma (HCC), MEOX2 predominantly exhibits tumour-suppressive properties. Studies have found that MEOX2 expression is lower in HCC tissues than in adjacent non-cancerous tissues, and its low expression correlates with higher Edmondson grade, vascular invasion, capsule invasion and shorter OS and disease-free survival (Zhou et al., 2012b). However, in the context of liver cancer stem cells (CSCs), the role of MEOX2 becomes complex. ABI2 protein recruits and interacts with MEOX2 to co-bind the promoter regions of KLF4 and NANOG and activate their transcription, thereby maintaining the CSC population in HCC, promoting tumour growth, metastasis and sorafenib resistance (Chen et al., 2022). This suggests that MEOX2 function may differ depending on cell state (differentiated cells versus stem cells). Thus, the net effect of MEOX2 in HCC is determined by the balance between its tumour-suppressive activity in differentiated cells and its pro-stemness function in CSCs.

In gastric cancer (GC), a functional single-nucleotide polymorphism (SNP), rs6489786, enhances binding of MEOX1 or MEOX2 to a distal enhancer, thereby upregulating the oncogenic lncRNA NRAV (Beck-Cormier et al., 2014). Additionally, MEOX2 may be involved in multidrug resistance in GC (Yan et al., 2014). These observations suggest that MEOX2 contributes to GC malignancy predominantly through non-coding RNA-mediated regulatory networks and drug resistance pathways, rather than through direct transcriptional regulation in this context.

In other digestive malignancies, MEOX2 has been implicated primarily as a prognostic or immune-related biomarker. In oesophageal squamous cell carcinoma (ESCC), bioinformatics analysis identified MEOX2 as a biomarker associated with tumour-associated macrophage infiltration. Its expression is closely correlated with the mRNA levels of colony-stimulating factor 1 (CSF-1) and its receptor CSF-1R, suggesting that MEOX2 may promote macrophage infiltration via the CSF-1/CSF-1R signalling axis (Wang et al., 2021). In colorectal cancer (CRC), MEOX2 is one of six key prognostic genes related to the tumour microenvironment, and its expression correlates with levels of immune cell infiltration (Liu Y. et al., 2021). In pancreatic intraductal papillary mucinous neoplasm (IPMN), downregulation of miR-130a may lead to upregulation of MEOX2, which is associated with high-risk IPMN (Permuth-Wey et al., 2015). In Wilms tumour (nephroblastoma), MEOX2 lies within a homozygous deletion region at 7p21 and is considered a candidate tumour suppressor gene (Ohshima et al., 2009; Blish et al., 2010). Collectively, these findings indicate that MEOX2 functions as a context-dependent regulator in digestive system cancers, with its net effect—tumour suppression or promotion—largely determined by cell differentiation status and the surrounding non-coding RNA regulatory network.

### 3.3 Thoracic tumours

In thoracic malignancies, MEOX2 exhibits a striking functional dichotomy: it acts as a potent oncogene in NSCLC, driving chemotherapy resistance and epigenetic dysregulation, while functioning predominantly as a tumour suppressor in breast cancer, where its loss correlates with poor prognosis and enhanced metastatic potential.

In non-small cell lung cancer (NSCLC), MEOX2 is oncogenic; its overexpression drives chemotherapy resistance and poor prognosis (Ávila-Moren et al., 2014). Mechanistic studies have revealed the importance of the MEOX2/GLI1 axis: MEOX2 directly binds to and activates the promoter of GLI1, jointly promoting resistance to cisplatin and EGFR tyrosine kinase inhibitors (TKIs) (e.g., erlotinib, osimertinib), and modulating the activation of EGFR/AKT/ERK signalling (Armas-López et al., 2017; Peralta-Arrieta et al., 2022). MEOX2 and GLI1 also induce epigenetic dysregulation of EGFR by reducing inhibitory marks (EZH2 and H3K27me3) and increasing activating histone marks (H3K27Ac/H3K4me3) at EGFR enhancer–promoter regions (Peralta-Arrieta et al., 2022). Furthermore, genome-wide association studies (GWAS) have identified that a genetic variant (rs10278557) within the MEOX2 gene is significantly associated with clinical stage and prognosis of lung adenocarcinoma (Frullanti et al., 2011). In lung squamous cell carcinoma, the downregulated lncRNA LINC01936 is predicted to potentially interact with epithelial–mesenchymal transition (EMT)-related genes including MEOX2 (Tian et al., 2023). In female lung adenocarcinoma, MEOX2 is included in a 12-gene prognostic signature (Sawada et al., 2022). SMARCB1-driven epigenetic alterations of EGFR-GLI1 are regulated by MEOX2 and GLI1 (Trejo-Villegas et al., 2025).

In breast cancer (BC), MEOX2 mainly functions as a tumour suppressor. Analysis of The Cancer Genome Atlas (TCGA) data and validation in clinical samples both show that MEOX2 is downregulated in BC tissues, and its low expression correlates with shorter OS of patients (Wang et al., 2022a; Wang et al., 2022b; Cai et al., 2022). Functionally, forced expression of MEOX2 inhibits BC cell proliferation, induces G0/G1 cell cycle arrest, suppresses metastatic potential and enhances chemosensitivity (Cai et al., 2022). The anti-cancer mechanisms involve negative regulation of the PI3K/AKT/mTOR and ERK1/2 signalling pathways (Cai et al., 2022). During metastasis, MEOX2 overexpression can inhibit BC cell adhesion to and transendothelial migration across lymphatic endothelial cells by targeting oxidative stress-induced RGS5 (Tang et al., 2025). In TNBC, the circ_0000190/miR-301a/MEOX2 axis has been identified as an important regulatory loop in which MEOX2 is a direct target of miR-301a; dysregulation of this axis promotes TNBC progression (Liu et al., 2025; Liu and Wang, 2021). Additionally, MEOX2 is a molecular feature of tumour-associated high endothelial venules (HEVs); its high expression correlates with extensive T-cell and B-cell infiltration, tertiary lymphoid structure formation and better survival in advanced BC (Sawada et al., 2022). MEOX2 has also been identified as a key angiogenesis-related gene (Wang et al., 2022b).

These contrasting roles in NSCLC and breast cancer highlight the extreme context dependency of MEOX2 function within thoracic malignancies, underscoring the necessity of tumour-type-specific strategies for therapeutic intervention.

### 3.4 Reproductive system tumours

In reproductive system tumours, MEOX2 primarily acts as a driver of malignancy. In ovarian cancer (OV), high MEOX2 expression is associated with cisplatin resistance, and silencing MEOX2 enhances chemosensitivity through E2F targets and DNA repair pathways (Wang F. et al., 2025). In cervical cancer (CC), the lncRNA MEOX2-AS1 promotes CC progression by sponging miR-143-3p to upregulate voltage-dependent anion channel 1 (VDAC1) (Liu X.-X. et al., 2021). Furthermore, MEOX2 has been identified as one of the key hub genes associated with lymph node metastasis in CC (Ding et al., 2023). These findings highlight the diversity of MEOX2’s oncogenic mechanisms in reproductive malignancies, ranging from direct transcriptional regulation of DNA repair genes to indirect modulation through non-coding RNA networks.

### 3.5 Other tumours

Beyond the major tumour types discussed above, MEOX2 has been implicated in several additional malignancies, where its roles range from tumour suppression to stromal heterogeneity and even chromosomal translocation-driven leukaemogenesis. In laryngeal cancer (LC), MEOX2 exerts a tumour-suppressive effect; overexpression of MEOX2 promotes apoptosis by inhibiting the PI3K/AKT pathway (Tian et al., 2018). In acute myeloid leukaemia (AML), a novel NUP98MEOX2 fusion gene has been discovered, providing direct evidence for the involvement of MEOX2 in haematological malignancies (Chonabayashi et al., 2026). Moreover, MEOX2 has been included in a complement system-related prognostic model (Liu and Liu, 2024). In the tumour microenvironment of prostate cancer, a cancer-associated fibroblast (CAF) subpopulation expressing MEOX2 (C3 MEOX2^+^) has been identified, highlighting the potential role of MEOX2 in tumour stromal heterogeneity (Ding et al., 2025). In melanoma, MEOX2 expression may correlate with disease progression, and its expression differs between intracranial and extracranial metastases (Kraft et al., 2024). Additionally, a functional SNP at the 10q24.33 risk locus affects MEOX2 binding (Cardinale et al., 2022). In kidney renal clear cell carcinoma (KIRC), a five-gene angiogenesis signature including MEOX2 effectively predicts patient prognosis (Zhou et al., 2025). These diverse associations underscore the pleiotropic nature of MEOX2 across distinct oncological contexts and suggest that its contribution to malignancy cannot be generalised, but rather must be evaluated on a tumour-type-specific basis.

## 4 Expression and function of MEOX2 in non-neoplastic diseases

**FIGURE 4:**
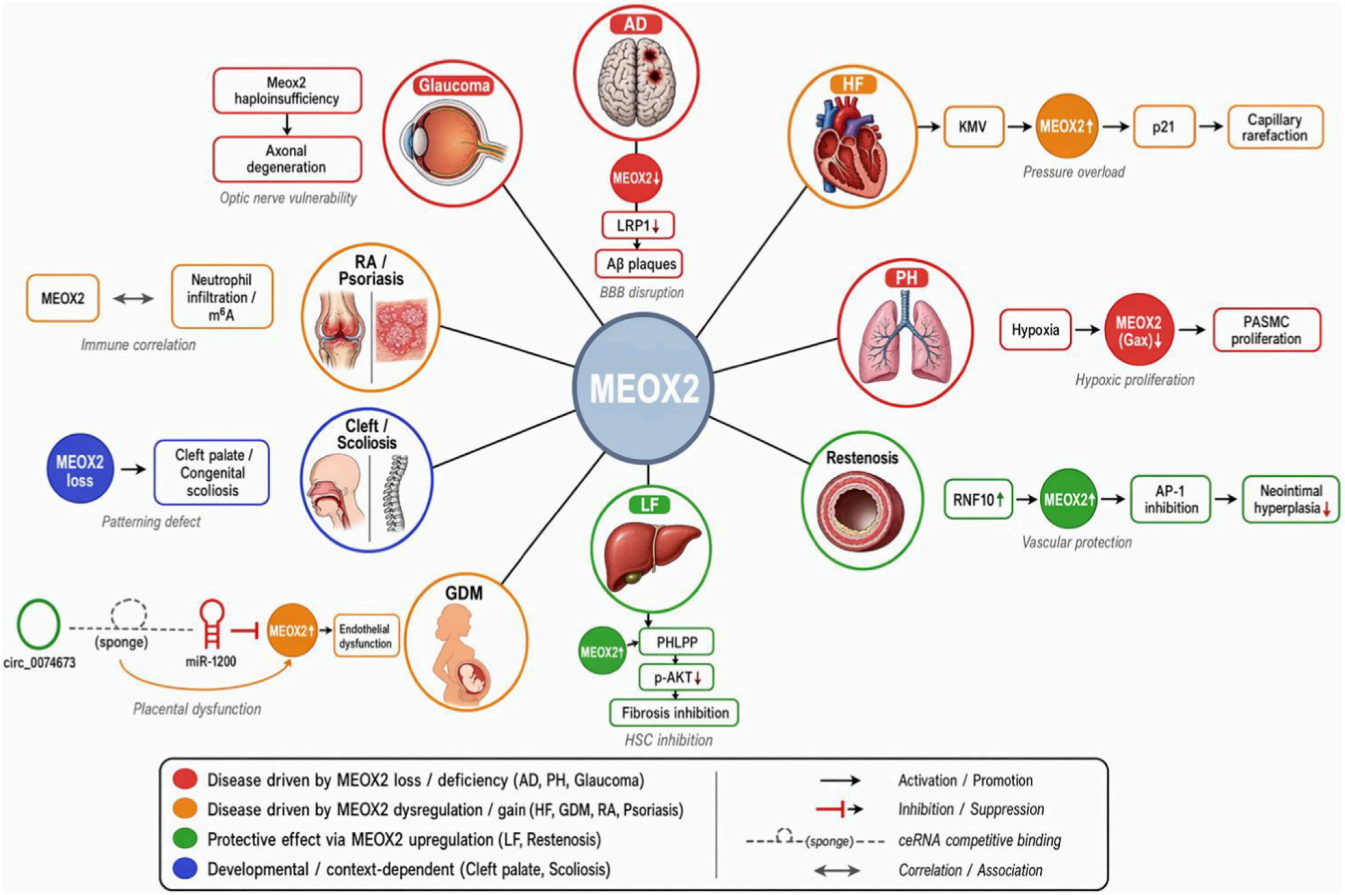
MEOX2 as a cross-disease regulatory node in non-neoplastic disorders. Schematic overview of MEOX2-mediated pathogenic and protective mechanisms across diverse non-neoplastic conditions. Diseases are color-coded according to the functional consequence of MEOX2 dysregulation: red, disorders driven by MEOX2 loss or deficiency (Alzheimer disease, glaucoma, pulmonary hypertension); orange, disorders driven by MEOX2 dysregulation or gain-of-function (heart failure, gestational diabetes mellitus, rheumatoid arthritis/psoriasis); green, conditions in which MEOX2 upregulation exerts a protective effect (liver fibrosis, restenosis); and blue, developmental or context-dependent disorders (cleft palate, congenital scoliosis). Key downstream effectors and molecular pathways are indicated for each disease entity. AD, Alzheimer disease; HF, heart failure; KMV, 3-methyl-2-oxovaleric acid; PH, pulmonary hypertension; LF, liver fibrosis; HSC, hepatic stellate cell; GDM, gestational diabetes mellitus; RA, rheumatoid arthritis; BBB, blood–brain barrier; LRP1, low-density lipoprotein receptor-related protein 1; Aβ, β-amyloid; PASMC, pulmonary artery smooth muscle cell; RNF10, RING finger protein 10; AP-1, activator protein-1; PHLPP, PH domain leucine-rich repeat protein phosphatase; circ_0074673, circular RNA 0074673.

Beyond cancer, dysregulation of MEOX2 expression is also widely involved in the pathogenesis of various non-neoplastic diseases. These encompass neurodegenerative disorders, cardiovascular diseases, metabolic and fibrotic conditions, developmental malformations and inflammatory immune-related diseases. Mirroring its dual role in cancer, the function of MEOX2 in non-neoplastic diseases also exhibits high context dependency: in pathological processes such as AD, pulmonary hypertension (PH) and cardiac fibrosis, its downregulation or loss of function is often a key driver of disease progression; whereas in scenarios like liver fibrosis and diabetic vascular complications, MEOX2 displays protective effects. These complex functional outputs are typically mediated through core signalling pathways such as PI3K/AKT, ERK and TGF-β, as well as epigenetic regulatory networks. Figure 4 provides an integrated overview of MEOX2-mediated pathogenic and protective mechanisms across these diverse non-neoplastic conditions. This section systematically reviews the expression patterns, molecular mechanisms and clinical associations of MEOX2 across various non-neoplastic diseases, summarised in Table 2.

**TABLE 2:** Mechanisms, molecular pathways and clinical associations of MEOX2 in various non-neoplastic diseases.

Disease category	Disease name	Mechanism of action	Molecular pathways	Clinical association	Ref.
Neurological disorders	Alzheimer disease (AD)	Decreased MEOX2 in brain endothelial cells → impaired angiogenesis, increased apoptosis, reduced Aβ clearance receptor LRP1; Meox2 haploinsufficiency exacerbates neuronal loss and plaque-associated microvessel reduction	Neurovascular dysfunction; LRP1-mediated Aβ clearance pathway	MEOX2 insufficiency directly linked to AD vascular pathology and neuronal loss; restoring MEOX2 has therapeutic potential; patient-derived iPSC model established	Wu et al. (2005), Soto et al. (2016), Wang et al. (2019), de la Torre, 2005; Bell (2012)
​	Pain perception	Altered nociception in Meox2 heterozygous mice; impaired action potential initiation after depolarisation	Voltage-gated sodium channel genes Scn9a and Scn11a downregulation	Maintains correct transcriptional program in primary afferent nociceptor neurons	Kokotović et al. (2022)
​	Dravet syndrome (DS)	Conditional Scn1a knockout using Meox2-Cre recapitulates DS phenotypes; interneuron-specific AAV-SCN1A gene therapy corrects epilepsy	GABAergic interneuron-specific SCN1A deficiency	Provides efficient animal model; Meox2-Cre-based gene replacement therapy corrects epileptic phenotype	Mich et al. (2025), Williams et al. (2019)
​	Glaucoma	Meox2 haploinsufficiency increases axonal damage without altering intraocular pressure; promotes axonal survival by controlling pressure-dependent vascular remodelling and neuroinflammation	Vascular remodelling; neuroinflammation	Potential neuroprotective target for delaying or preventing glaucoma	Buchanan et al. (2019)
​	Huntington disease (HD)	HTT loss-of-function leads to significant upregulation of MEOX2 (early transcriptional dysregulation)	Transcription factor network dysregulation	Suggests MEOX2 involvement in early molecular pathology of HD	Kozłowska et al. (2025)
​	Autism spectrum disorder (ASD)	Identified as a potential candidate risk gene through PPI network topology analysis	Protein-protein interaction network	May contribute to ASD genetic risk	Remori et al. (2025)
Cardiovascular diseases	Coronary heart disease (CHD)	MEOX2 GTCCGC haplotype carriers have significantly increased risk of CHD, myocardial infarction, revascularisation, ischaemic cardiomyopathy	Genetic variation	Strong predictor, with population attributable risk similar to smoking (18.7% vs. 16.2%)	Yang et al. (2015)
​	Cardiac fibrosis	Meox2 expression decreases during fibroblast-to-myofibroblast transition; Meox2 overexpression reverts myofibroblast to fibroblast phenotype	Ski-Zeb2-Meox2 pathway	Regulates cardiac myofibroblast phenotype; provides new target for treating myocardial fibrosis	Cunnington et al. (2014)
​	Heart failure (HF)	Pressure overload → hypertrophic cardiomyocytes secrete KMV → increased Meox2 expression/nuclear accumulation in endothelial cells → Meox2 binds p21 promoter → triggers endothelial senescence, impaired angiogenesis → capillary rarefaction	KMV-mediated cardiomyocyte-endothelial cell signalling; p21 senescence pathway	Targeting KMV-Meox2 axis has therapeutic potential; OGN as HF diagnostic marker is regulated by MEOX2	Gao et al. (2025), Zhu et al. (2024)
​	Vascular restenosis	RNF10 upregulates MEOX2 and inhibits AP-1, reducing neointimal hyperplasia	RNF10/AP-1/Meox2 axis	RNF10 is a potential therapeutic target for vascular restenosis	Li et al. (2019)
​	Chemotherapy-induced cardiotoxicity	Pirarubicin (THP)-induced cardiac inflammation and apoptosis linked to dysregulation of RNF10/AP-1/Meox2 axis	RNF10/AP-1/Meox2 signalling axis	RNF10 may be a novel target for treating THP cardiotoxicity	Shi et al. (2023), Duan et al. (2023)
​	Pulmonary hypertension (PH)	Hypoxia downregulates MEOX2 (Gax) in PASMCs; Gax overexpression inhibits hypoxia-induced PASMC proliferation (via ERK1/2) and induces apoptosis (via Bcl-2/Bax)	ERK1/2 pathway; Bcl-2/Bax apoptosis pathway	Gax gene therapy counteracts hypoxic PH development	Xia et al. (2011), Lin and Mequanint (2015)
Metabolic and fibrotic diseases	Liver fibrosis (LF)	MEOX2 overexpression binds PHLPP promoter and upregulates its transcription → inhibits AKT phosphorylation → inhibits HSC proliferation, slows fibrosis progression	PI3K/AKT pathway (inhibition)	MEOX2 is a protective factor and potential therapeutic target for liver fibrosis	Wang et al. (2024)
​	Gestational diabetes mellitus (GDM)	Placental exosomal circ_0074673 sponges miR-1200 to upregulate MEOX2 → inhibits umbilical vein endothelial cell function; NAT10 stabilises MEOX2 mRNA via ac4C modification → inhibits endothelial function	circRNA/miRNA/MEOX2 axis; ac4C RNA modification	circ_0074673 and NAT10 are potential therapeutic targets for GDM	Huang et al. (2021), Huang et al. (2025)
​	Diabetic cardiac fatty acid metabolism	Hyperglycaemia promotes LPL release from cardiomyocytes via TGF-β signalling; TGF-β also promotes MEOX2 nuclear translocation in endothelial cells, increasing GPIHBP1 expression and accelerating LPL transport	TGF-β/MEOX2/GPIHBP1 axis	Molecular mechanism for enhanced fatty acid utilisation in the diabetic heart	Chiu et al. (2018)
​	Adipose metabolism	Gax inhibits IGF-1-induced proliferation and differentiation of perivascular adipocytes; MEOX2 is a molecular marker distinguishing white/beige adipocytes from brown adipocytes/myocytes	PI3K/Akt, ERK, FAK/Pyk2 pathways (inhibition)	Potential target for intervention in adiposity-related cardiovascular diseases	Petrovic et al. (2010), Liu et al. (2015), Liu et al. (2014), Jiang et al. (2018), Waldén et al. (2010)
​	Skeletal muscle development and differentiation	Meox2 loss leads to reduced fetal myoblast number and increased oxidative muscle fibres; miR-148a-3p targets Meox2 to regulate differentiation and apoptosis	PI3K/AKT pathway (miR-148a-3p/Meox2)	Regulates skeletal muscle satellite cell differentiation and apoptosis	Otto et al. (2010), Yin et al. (2020), Goljanek-Whysall et al. (2012)
Developmental and congenital disorders	Cleft palate (non-syndromic cleft palate only)	Key gene for maintaining post-fusion palatal adhesion; Meox2 loss-of-function causes post-fusion cleft palate; critical regulator of posterior palate development	TGFβ3 signalling pathway; Msx1 suppresses Meox2 to regulate anteroposterior patterning	SNP rs2237493 significantly associated in Vietnamese females; single-cell multi-omics identifies MEOX2 as key regulator of posterior palate development	Jin and Ding (2006), Li and Ding (2007), Tran et al. (2018), Yan et al. (2024), Neupane et al. (2018), Zhu et al. (2022), Shi et al. (2019), Fantauzzo and Soriano (2017), Smith et al. (2012), Ozturk et al. (2013), Hampl et al. (2020)
​	Congenital scoliosis (CS)	Candidate gene in TBX6-mediated somitogenesis pathway	Somitogenesis genetic network	Included in CS-related genetic network analyses	Yang et al. (2020a)
​	Neural tube defects (NTDs)	Candidate gene on chromosome 7; involved in vascular development and neural tube closure	Vascular development	Candidate gene identified by genome-wide linkage analysis	Rampersaud et al. (2005)
​	Placental development	Meox2-Cre used for conditional knockout of multiple placenta-related genes (*Pkd1/2*, Csf1, Dlx3, IP3R); Meox2 downregulated in *Peg11/Rtl1* KO placenta	Placental angiogenesis; oxidative stress	Important tool gene for studying placental function	Yang et al. (2020b), Yang et al. (2020c), Garcia-Gonzalez et al. (2010), Harris et al. (2012), Clark et al. (2012), Kitazawa et al. (2017)
​	Bone healing	Meox2 knockdown promotes bone regeneration	Osteoblast activation; inflammation regulation	Potential target for gene therapy to promote bone regeneration	Kim et al. (2020)
Other diseases	Rheumatoid arthritis (RA)	Altered MEOX2 expression in synovial tissues correlates with immune cell infiltration (neutrophils)	Immune infiltration; NET-related genes	Diagnostic biomarker (together with LSP1, GNLY)	Sheng et al. (2025), Yu et al. (2021), Comertpay and Gov (2020)
​	Psoriasis	Differentially expressed gene associated with m^6^A methylation	m^6^A RNA modification	Associated with cell proliferation and neuroregulation dysregulation	Gan et al. (2024)
​	Wound healing and scar formation	Downregulation of MEOX2 promotes fibroblast reprogramming into myofibroblasts; miR-1908 inhibits Ski to upregulate Meox2	Transcription factor network; miR-1908/Ski/Meox2 axis	Key transcription factor controlling fibroblast differentiation; therapeutic target for scar formation	Noizet et al. (2016), Xie et al. (2016)
​	Kidney disease (declining renal function)	GWAS identified GALNT11 and CDH23 loci near MEOX2 associated with declining renal function	Genetic variation	Loci associated with renal function decline in European populations	Gorski et al. (2015)
​	Hutchinson–Gilford progeria syndrome (HGPS)	MEOX2 is among the most significantly downregulated genes; involved in maintaining mesodermal/mesenchymal tissue homeostasis	Transcriptional dysregulation	May explain widespread developmental deficits and accelerated atherosclerosis in HGPS	Csoka et al. (2004)
​	Influenza virus host interaction	MEOX2 is among the host proteins with the most interactions with viral proteins	Virus-host protein interaction network	Potential anti-viral target	Farooq et al. (2020)

### 4.1 Neurological disorders

MEOX2 dysfunction is intimately linked to a spectrum of neurological disorders, where its roles range from vascular pathology in neurodegeneration to synaptic dysfunction in epilepsy and genetic susceptibility in neurodevelopmental conditions.

In Alzheimer disease (AD), the neurovascular dysfunction hypothesis for MEOX2 has gained broad support. Studies have shown that MEOX2 expression is decreased in brain endothelial cells (BECs) of patients with AD (Wu et al., 2005). Functionally, restoring MEOX2 expression stimulates angiogenesis, inhibits apoptosis and increases the level of LRP1, a major Aβ clearance receptor. In mice, Meox2 deletion leads to reduced brain capillary density and resting cerebral blood flow, loss of the angiogenic response to brain hypoxia, and impaired brain Aβ clearance due to reduced LRP1 levels (Wu et al., 2005). Genetic studies support this hypothesis: in APP/PS1 AD model mice, Meox2 haploinsufficiency exacerbates neuronal loss, especially in areas with plaque deposition, and is accompanied by a significant reduction in plaque-associated microvessels (Soto et al., 2016). These findings directly link MEOX2 insufficiency to vascular pathology and neuronal loss in AD, making it a potential therapeutic target (de la Torre, 2005; Bell, 2012). The establishment of a patient-derived iPSC line (ZZUi0013-A) carrying a novel MEOX2 mutation provides a valuable model for studying the pathological mechanism of MEOX2 in AD (Wang et al., 2019). In cerebrovascular gene studies, MEOX2 is a key gene related to AD pathology (de la Torre, 2005). These findings link MEOX2 deficiency in cerebral vasculature to AD neurovascular pathology, positioning it as a therapeutic target.

Beyond AD, MEOX2 dysfunction has been implicated in several other neurological conditions with distinct pathophysiological mechanisms. In Dravet syndrome (DS), a severe epileptic encephalopathy, conditional Scn1a knockout using the Meox2-Cre driver has successfully recapitulated the seizure phenotypes, and interneuron-specific dual-AAV SCN1A gene replacement based on Meox2-Cre has shown therapeutic efficacy in mouse models (Mich et al., 2025; Williams et al., 2019). In the domain of pain perception, Meox2 heterozygous mice exhibit altered nociceptive function associated with reduced expression of voltage-gated sodium channel genes Scn9a and Scn11a in primary afferent neurons, revealing a previously unrecognized role for MEOX2 in maintaining the transcriptional program of nociceptors (Kokotović et al., 2022). Additionally, systems biology analyses have nominated MEOX2 as a potential candidate risk gene for autism spectrum disorder (ASD) through protein-protein interaction network topology (Remori et al., 2025). Collectively, these findings extend the functional repertoire of MEOX2 beyond vascular biology into neuronal excitability and neurodevelopmental risk.

In glaucoma research, transcriptomic analysis using the DBA/2J mouse model identified Meox2 as a potential upstream regulator of early gene expression changes in the optic nerve head. While Meox2 haploinsufficiency did not affect intraocular pressure elevation, it significantly increased the number of eyes with axonal damage. Mechanistically, Meox2 may promote axonal survival by controlling intraocular pressure-dependent vascular remodelling and neuroinflammation (Buchanan et al., 2019).

In the early stages of Huntington disease (HD), transcriptomic analysis revealed that MEOX2 is one of the key transcription factors significantly upregulated upon loss of HTT function, suggesting a role in the transcriptional dysregulation network early in the disease (Kozłowska et al., 2025).

Together, these neurological associations establish MEOX2 as a key regulator spanning both vascular and neuronal compartments, with its dysfunction contributing to pathogenesis through distinct mechanisms in each context.

### 4.2 Cardiovascular diseases

In the cardiovascular system, MEOX2 plays a pivotal role in maintaining vascular homeostasis, and its dysregulation—whether through genetic variation, epigenetic silencing or post-translational modification—contributes to a spectrum of pathological conditions including ischaemic heart disease, fibrosis and pulmonary hypertension.

Epidemiological studies indicate that genetic variants of MEOX2 are strong predictors of coronary heart disease (CHD). In the Flemish population cohort, carriers of the MEOX2 GTCCGC haplotype had a significantly increased risk of CHD, myocardial infarction, coronary revascularisation and ischaemic cardiomyopathy (Yang et al., 2015).

In cardiac fibrosis, the Ski–Zeb2–Meox2 pathway has been revealed as a novel mechanism regulating the phenotype of cardiac myofibroblasts. During fibroblast-to-myofibroblast transition, Meox2 expression decreases, whereas overexpression of Meox2 reverts myofibroblasts towards a fibroblast phenotype (Cunnington et al., 2014). Similarly, in hypertrophic scars, Ski can upregulate Meox2 expression to inhibit Zeb2, thereby reducing the TGF-β1-induced myofibroblast phenotype (Liu et al., 2020).

In the development of heart failure (HF), recent studies have uncovered a key role for MEOX2 in capillary rarefaction. Under pressure overload, hypertrophic cardiomyocytes secrete the metabolite 3-methyl-2-oxovaleric acid (KMV), which increases Meox2 expression and nuclear accumulation in endothelial cells. Nuclear Meox2 directly binds to the promoter of the senescence-associated gene p21, triggering endothelial senescence and impaired angiogenesis, ultimately leading to capillary rarefaction and promoting HF (Gao et al., 2025). Additionally, OGN, a diagnostic biomarker for HF, is regulated in a manner involving MEOX2 (Zhu et al., 2024). These findings highlight a novel cardiomyocyte-to-endothelial cell signalling axis in which MEOX2 serves as a critical mediator of capillary rarefaction and cardiac dysfunction.

In the pathology of vascular restenosis, RNF10 has been shown to inhibit excessive proliferation of vascular smooth muscle cells by regulating the MEOX2/AP-1 axis. Overexpression of RNF10 upregulates MEOX2 and inhibits AP-1, thereby reducing neointimal hyperplasia (Li et al., 2019).

In related vascular stress contexts, chemotherapy-induced cardiotoxicity has also been linked to dysregulation of the RNF10/AP-1/Meox2 axis, as pirarubicin (THP)-induced cardiac inflammation and apoptosis are modulated through this signalling cascade (Shi et al., 2023; Duan et al., 2023). In pulmonary hypertension (PH), hypoxia downregulates MEOX2 (Gax) expression in pulmonary artery smooth muscle cells (PASMCs); conversely, adenoviral-mediated Gax overexpression inhibits hypoxia-induced PASMC proliferation—putatively via the ERK1/2 pathway—and induces apoptosis through the Bcl-2/Bax axis, thereby counteracting hypoxic PH development (Xia et al., 2011; Lin and Mequanint, 2015). These observations position MEOX2 as a common mediator of vascular remodelling across distinct haemodynamic and pharmacological insults.

These cardiovascular findings collectively position MEOX2 as a critical node connecting metabolic stress, vascular remodelling and cardiac dysfunction, with therapeutic implications for both ischaemic and pressure-overload heart disease.

### 4.3 Metabolic and fibrotic diseases

In metabolic and fibrotic diseases, MEOX2 exhibits a predominantly protective role, counteracting pathological processes through inhibition of PI3K/AKT signalling in hepatic stellate cells and modulation of lipid metabolism in adipose and skeletal muscle tissues.

In liver fibrosis (LF), MEOX2 shows a protective effect. Comparison of differentially expressed genes between adult liver stem/progenitor cells and hepatic stellate cells (HSCs) revealed that MEOX2 is upregulated in HSCs. Functionally, overexpression of MEOX2 significantly inhibits HSC viability and proliferation. Mechanistically, MEOX2 binds to the promoter of PHLPP and upregulates its transcription, thereby inhibiting AKT phosphorylation and slowing the progression of liver fibrosis (Wang et al., 2024).

In diabetes mellitus and its complications, the role of MEOX2 has been revealed from multiple angles. In gestational diabetes mellitus, placental exosomal circ_0074673 upregulates MEOX2 by sponging miR-1200, inhibiting umbilical vein endothelial cell proliferation, migration and angiogenesis (Huang et al., 2021). Another study found that NAT10, an enzyme mediating RNA ac4C modification, is upregulated in GDM placentas; it stabilises the MEOX2 transcript by increasing ac4C modification at specific sites in the MEOX2 mRNA, thereby inhibiting endothelial cell function (Huang et al., 2025). In the fatty acid metabolism of the diabetic heart, hyperglycaemia promotes the release of lipoprotein lipase (LPL) from cardiomyocytes via TGF-β signalling; concurrently, TGF-β signalling also promotes nuclear translocation of MEOX2 in endothelial cells, increasing GPIHBP1 expression and accelerating the transport of LPL to the vascular lumen, thereby enhancing fatty acid utilisation by the heart (Chiu et al., 2018). In endothelial colony-forming cells exposed to intrauterine diabetes, MEOX2 promotes migration (Gohn et al., 2017).

In adipose metabolism, MEOX2 (Gax) has been shown to inhibit insulin-like growth factor-1 (IGF-1)-induced proliferation, differentiation and biological functions of perivascular adipocytes (PVACs) and preadipocytes (3T3-F442A). The mechanism involves inhibition of IGF-1-mediated activation of PI3K/Akt, ERK and FAK/Pyk2 signalling pathways (Liu et al., 2015; Liu et al., 2014; Jiang et al., 2018). Interestingly, in epididymal white adipocytes following long-term PPARγ activation, a population of ‘beige’ adipocytes with thermogenic capacity but molecularly distinct from classical brown adipocytes emerges; these cells do not express the brown fat signature genes Zic1, Lhx8 or Meox2, indicating that MEOX2 is a molecular marker distinguishing white/beige adipocytes from brown adipocytes/myocytes (Petrovic et al., 2010; Waldén et al., 2010). In studies of adipogenesis from sheep adipose-derived stromal vascular fraction, circINSR acts via the miR-152/MEOX2 axis (Zhao et al., 2023).

### 4.4 Developmental and congenital disorders

In developmental biology, MEOX2 is a critical determinant of axial patterning and organogenesis, with its most extensively characterised roles in palatogenesis, somitogenesis and placental development.

In the pathogenesis of cleft palate, MEOX2 is one of the most intensively studied genes. It has been identified as a key regulator of posterior secondary palate development in mice (Yan et al., 2024; Smith et al., 2012; Hampl et al., 2020). Its expression is dynamic: at E12.5, it occupies most of the palatal shelf, then gradually recedes to the posterior region, while the expression domain of the anterior marker Shox2 expands accordingly; this transition may involve cell fate reprogramming (Li and Ding, 2007). Loss of Meox2 function leads to a unique post-fusion cleft palate, where the palatal shelves fuse but subsequently rupture, providing the first evidence for genes required to maintain post-fusion palatal adhesion (Jin and Ding, 2006). In the cleft palate model of TGFβ3-knockout mice, Meox2 is one of the few known cleft palate-related genes whose expression is significantly altered (Ozturk et al., 2013). Human genetic studies have shown that the MEOX2 SNP rs2237493 is significantly associated with non-syndromic cleft palate only in Vietnamese females (Tran et al., 2018). In palatal mucosal differentiation, Meox2 regulates epithelial differentiation (Neupane et al., 2018). In mouse embryonic palatal mesenchymal cells, Meox2 is a marker of stemness maintenance (Shi et al., 2019; Fantauzzo and Soriano, 2017). Msx1 regulates palatal anteroposterior patterning by suppressing Meox2 (Zhu et al., 2022). Single-cell multi-omics decoding has revealed MEOX2 as an important regulator of posterior palate development (Yan et al., 2024).

Beyond palatogenesis, MEOX2 participates in multiple other developmental programs. In congenital scoliosis (CS), MEOX2 has been nominated as a candidate gene within the TBX6-mediated somitogenesis pathway, although large-scale sequencing has not identified high-frequency mutations in MEOX2 itself (Yang Y. et al., 2020). In neural tube defects (NTDs), linkage analysis has placed MEOX2 within a region of interest on chromosome 7, consistent with its potential role in vascular development and neural tube closure (Rampersaud et al., 2005). In placental biology, Meox2-Cre has been extensively employed for conditional knockout of multiple placenta-related genes, including Pkd1/2, Csf1, Dlx3 and IP3R, underscoring its utility as a genetic tool in this organ (Yang F. et al., 2020; Yang L. et al., 2020; Garcia-Gonzalez et al., 2010; Harris et al., 2012; Clark et al., 2012; Kitazawa et al., 2017); conversely, Peg11/Rtl1 knockout placentas exhibit downregulated Meox2 expression, linking MEOX2 to placental vascular integrity (Kitazawa et al., 2017). In bone healing, Meox2 knockdown has been reported to promote bone regeneration, suggesting a potential gene-therapy target for skeletal repair (Kim et al., 2020). Collectively, these diverse developmental roles highlight MEOX2 as a pleiotropic regulator of morphogenesis beyond the palate.

In skeletal muscle development, Meox2 loss leads to a reduction in fetal myoblast number and a compensatory increase in oxidative muscle fibres (Otto et al., 2010). This process is subject to multilayered post-transcriptional regulation by multiple miRNAs: in chicken skeletal muscle satellite cells, miR-148a-3p regulates differentiation and apoptosis by targeting Meox2 (Yin et al., 2020); in C2C12 myoblasts, miR-1 and miR-206 similarly target Meox2 during myogenic differentiation (Goljanek-Whysall et al., 2012). Additionally, Meox2-Cre-mediated Nle1 deletion disrupts axial skeleton formation (Beck-Cormier et al., 2014), and adrenomedullin 2 (AM2) upregulates MEOX2 to inhibit retinal neovascularisation (Kakihara et al., 2023), further supporting the pleiotropic role of MEOX2 in morphogenesis. In other developmental processes, such as cloaca/perineal muscle development, studies suggest that these muscles originate from hindlimb muscles, and their absence in Met mutants implies that their developmental mechanisms are similar to those of limb muscles, with MEOX2, as an important regulator of limb muscle development, potentially being indirectly involved (Valasek et al., 2005).

These developmental studies underscore MEOX2’s evolutionary conserved role in morphogenesis and establish a foundation for understanding how its dysregulation contributes to congenital anomalies.

### 4.5 Other diseases

In inflammatory and fibrotic conditions, MEOX2 dysregulation has emerged as a recurrent theme. In rheumatoid arthritis (RA), multiple bioinformatics analyses have identified MEOX2 as a diagnostic biomarker, with its altered expression in synovial tissues correlating with immune cell infiltration, particularly neutrophils, and neutrophil extracellular trap (NET)-related gene signatures (Sheng et al., 2025; Yu et al., 2021; Comertpay and Gov, 2020). In psoriasis, MEOX2 has been flagged as a differentially expressed gene associated with m^6^A RNA methylation, suggesting a potential link to aberrant cell proliferation and neuroregulatory dysfunction in psoriatic lesions (Gan et al., 2024). In wound healing and scar formation, MEOX2 functions as a key transcription factor controlling fibroblast-to-myofibroblast differentiation; its downregulation promotes this transition, while miR-1908 indirectly upregulates Meox2 by targeting Ski, thereby modulating fibrotic outcomes in burn scars (Noizet et al., 2016; Xie et al., 2016).

Beyond inflammation, MEOX2 has been linked to systemic and genetic disorders. In genome-wide association studies of kidney function, loci near MEOX2 (GALNT11 and CDH23) have been associated with declining renal function in European populations (Gorski et al., 2015). In Hutchinson–Gilford progeria syndrome (HGPS), MEOX2 is among the most significantly downregulated genes in patient fibroblasts, consistent with its role in maintaining mesodermal/mesenchymal tissue homeostasis and potentially explaining the accelerated atherosclerosis and developmental defects seen in this disorder (Csoka et al., 2004). Additionally, in influenza A virus host-interaction networks, MEOX2 has been identified as a viral protein-interacting host factor, raising the possibility of its involvement in antiviral responses (Farooq et al., 2020; Lee et al., 2017). These diverse associations underscore the broad pathophysiological reach of MEOX2 across immune-mediated, degenerative and infectious contexts.

## 5 Clinical translational potential of MEOX2

The preceding sections have systematically delineated the complex and context-dependent expression patterns and molecular mechanisms of MEOX2 in numerous neoplastic and non-neoplastic diseases. Importantly, whether in its oncogenic or tumour-suppressive roles, the expression level of MEOX2 shows significant correlation with patient clinicopathological characteristics and survival outcomes, providing a solid data foundation for its use as a diagnostic and prognostic biomarker. Concurrently, as a transcriptional regulatory hub, multiple nodes within its upstream and downstream signalling pathways and non-coding RNA networks hold potential for pharmacological intervention.

### 5.1 As a diagnostic and prognostic biomarker

MEOX2 expression has robust prognostic value in several cancers, including glioma, breast cancer, HCC, lung cancer and ovarian cancer (Schönrock et al., 2022; Tachon et al., 2019; Wang et al., 2022a; Cai et al., 2022; Zhou et al., 2012b; Frullanti et al., 2011; Wang F. et al., 2025). This positions MEOX2 as a potential tool for molecular stratification and prognosis assessment in these tumours.

In terms of ancillary pathological diagnosis, IHC detection of MEOX2 shows practical value. Studies indicate that combined detection of MEOX2, SOX11, INSM1 and EGFR in limited biopsy samples can effectively distinguish GBM cells from reactive gliosis, improving diagnostic accuracy (Soukup et al., 2023).

Furthermore, prognostic signature models based on MEOX2 combined with other genes are being continually developed and refined. For example, in LGG, a palmitoylation-related five-gene signature (CHI3L1, IGFBP2, MEOX2, EMILIN3, SFRP2) and an immune microenvironment-related six-gene signature (MEOX2, PHYHIP, RBBP8, ST18, TCF12, THRB) both demonstrate robust prognostic prediction capabilities (Wu et al., 2025; Wang Z. et al., 2025). In KIRC, a five-gene angiogenesis signature (MEOX2, PLG, PROX1, TEK, TIMP1) effectively predicts patient prognosis (Zhou et al., 2025). In AML, a complement system-related four-gene signature including MEOX2 also has prognostic value (Liu and Liu, 2024). In female lung adenocarcinoma, a 12-gene signature including MEOX2 predicts prognosis (Sawada et al., 2022).

### 5.2 Development strategies as a therapeutic target

Direct Targeting of MEOX2: For MEOX2-driven tumours (e.g., GBM, lung cancer), direct inhibition of its transcriptional activity or promotion of its degradation is an ideal strategy. Using genetic and pharmacologic approaches, Lu et al. identified FDA-approved drugs targeting MEOX2 (Lu et al., 2025). Virtual molecular screening and subsequent dose-response validation revealed that nilotinib and DHEC (dihydroergocryptine) have inhibitory effects on MEOX2. Mechanistically, these drugs suppress the MEOX2-NOTCH axis in classical (CL) GSCs. Notably, MEOX2 knockdown decreased the sensitivity of CL GSCs to nilotinib and DHEC, further confirming the specificity of these agents. Combined targeting of CL GSCs (via MEOX2 inhibition) and mesenchymal (MES) GSCs (via SRGN inhibition with paliperidone and risperidone) demonstrated enhanced efficacy both *in vitro* and *in vivo* (Lu et al., 2025). However, as transcription factors are traditionally considered ‘undruggable’ targets, developing highly selective, potent small-molecule inhibitors remains challenging. New technologies such as PROteolysis TArgeting Chimeras (PROTACs) may offer new avenues for targeting MEOX2.

Combination Therapy Strategies: Given that MEOX2 typically acts as a signalling pathway hub, intervening in its upstream or downstream pathways in combination may be more feasible and effective. In GBM, co-targeting MEOX2 (enriched in the proneural GSC state) and SRGN (enriched in the mesenchymal GSC state) eliminated heterogeneous GSC populations more effectively than single targeting (Lu et al., 2025). In lung cancer, combining inhibitors of the MEOX2/GLI1 axis with EGFR-TKIs or platinum-based chemotherapy holds promise for overcoming resistance to existing treatments (Armas-López et al., 2017; Peralta-Arrieta et al., 2022).

Indirect Intervention via Regulatory Networks (1) miRNA-based therapy: Using miRNA mimics (e.g., miR-301a mimic to target oncogenic MEOX2): or inhibitors (e.g., miR-130a inhibitor to upregulate tumour-suppressive MEOX2) shows preclinical potential for modulating MEOX2 expression and therapeutic efficacy in BC, HCC and other diseases (Liu et al., 2025; Zhou et al., 2012a). Targeting the MEOX2-AS1/miR-143-3p axis may also represent a therapeutic strategy for CC (Liu X.-X. et al., 2021). In pulmonary artery endothelial cells, framework nucleic acids regulate Meox2 by targeting miR-152 (You et al., 2022). (2) Targeting upstream regulators: For example, in cardiovascular disease and liver fibrosis, activating RNF10 or intervening in TGF-β signalling can indirectly upregulate MEOX2, exerting protective effects (Wang et al., 2024; Li et al., 2019). In diabetic cardiac complications, modulating the TGF-β/MEOX2 axis may ameliorate metabolic imbalance (Chiu et al., 2018). In AD, restoring MEOX2 expression holds therapeutic potential (Wu et al., 2005). (3) Targeting downstream effector pathways: Even if direct MEOX2 targeting is not feasible, inhibiting its activated downstream pathways (e.g., PI3K/AKT, ERK, Hh) remains a currently viable therapeutic option, particularly in tumours with high MEOX2 expression (Schönrock et al., 2022; Peralta-Arrieta et al., 2022; Cai et al., 2022). (4) Gene Therapy and Cell Therapy: In monogenic disorders or diseases involving loss of function, restoring MEOX2 function is therapeutically promising. For example, in the vascular pathology of AD, theoretically restoring MEOX2 expression in brain endothelial cells could improve blood–brain barrier function and Aβ clearance (Wu et al., 2005). In DS, Meox2-Cre-mediated, interneuron-specific SCN1A gene replacement has been successfully achieved in mouse models, demonstrating the potential of precision gene therapy (Mich et al., 2025; Williams et al., 2019). Furthermore, in cell therapy, hypoxic preconditioning of HUVECs upregulates a set of genes including MEOX2, enhancing their migration and neurovascular protective capacity, offering a new strategy for treating neonatal hypoxic-ischaemic encephalopathy (Lee et al., 2018).

In summary, MEOX2 exhibits multi-dimensional potential for clinical translation. Its expression levels correlate significantly with patient prognosis in GBM, BC, lung cancer, OV and other malignancies. Multi-gene models incorporating MEOX2 enhance risk stratification, and IHC detection aids ancillary pathological diagnosis. Direct targeting, combination strategies and indirect interventions via miRNAs, lncRNAs and gene editing are advancing. Gene replacement and cell therapies show preliminary efficacy in AD, DS and neonatal brain ischaemia. These translational strategies are summarised in Table 3.

**TABLE 3:** Clinical translational strategies for MEOX2: diagnostic/prognostic biomarkers, therapeutic targets and intervention methods.

Application area	Specific strategy/Biomarker	Mechanism/Target	Clinical association	Evidence type/Model source	Developmental stage	Ref.
Diagnostic/prognostic biomarkers	MEOX2 mRNA or protein expression level	Acts as an independent prognostic predictor	Significantly correlated with overall survival and progression-free survival in gliomas (especially GBM and LGG), breast cancer, HCC, lung cancer, and ovarian cancer	Human tissue specimens (IHC, qPCR, Western blot) and retrospective analyses of public clinical databases (TCGA, CGGA, GEO)	Clinical association validation (retrospective cohort studies)	Schönrock et al. (2022), Tachon et al. (2019), Wang et al. (2022a), Cai et al. (2022), Zhou et al. (2012b), Frullanti et al. (2011), Wang et al. (2025b)
Diagnostic/prognostic biomarkers	IHC panel (MEOX2 + SOX11 + INSM1 + EGFR)	Distinguishes GBM cells from reactive gliosis	Improves diagnostic accuracy in limited biopsy samples	Human tissue specimens (tissue microarrays, IHC)	Clinical ancillary diagnostic application (validated in limited biopsies)	Soukup et al. (2023)
Diagnostic/prognostic biomarkers	Palmitoylation-related 5-gene signature (CHI3L1, IGFBP2, MEOX2, EMILIN3, SFRP2)	Palmitoylation-associated pathways	Risk stratification for LGG prognosis	Bioinformatics predictions (multi-omics) + *in vitro* validation (siRNA knockdown of IGFBP2)	Model construction and retrospective validation (prospective cohort needed)	Wang et al. (2025a)
Diagnostic/prognostic biomarkers	Immune microenvironment-related 6-gene signature (MEOX2, PHYHIP, RBBP8, ST18, TCF12, THRB)	Tumour microenvironment immune infiltration	Prognostic prediction for GBM	Bioinformatics analysis (TCGA, GEO)	Model construction and retrospective validation	Wu et al. (2025)
Diagnostic/prognostic biomarkers	Angiogenesis-related 5-gene signature (MEOX2, PLG, PROX1, TEK, TIMP1)	Angiogenesis-associated pathways	Prognostic prediction for KIRC	Bioinformatics analysis (TCGA) + single-cell data	Model construction and retrospective validation	Zhou et al. (2025)
Diagnostic/prognostic biomarkers	Complement system-related 4-gene signature (MEOX2, IGFBP5, CH25H, RAB3B)	Complement system-associated pathways	Prognostic prediction for AML	Bioinformatics analysis (TCGA-AML, GSE37642)	Model construction and retrospective validation	Liu and Liu (2024)
Diagnostic/prognostic biomarkers	12-gene signature (including MEOX2) for female lung adenocarcinoma	Sex-specific oncogenic genes	Prognostic prediction specific to female lung adenocarcinoma	Bioinformatics analysis (TCGA, GSE72094) + human tissue IHC (CABLES1)	Model construction and retrospective validation	Feng et al. (2024)
Direct targeting of MEOX2	Small-molecule inhibitors (nilotinib, DHEC)	Inhibit MEOX2 transcriptional activity or promote degradation	Anti-tumour efficacy in MEOX2-high tumours (GBM, lung cancer)	*In vitro* (GSC sphere formation, dose-response, MEOX2 KD sensitivity) + *in vivo* (intracranial xenograft mice)	Preclinical (*in vivo* proof-of-concept completed)	Lu et al. (2025)
Direct targeting of MEOX2	PROTAC and other protein degradation technologies	Targeted degradation of MEOX2 protein	New strategy to overcome the “undruggable” challenge of transcription factors	Discussion of emerging technology, no experimental data available	Theoretical/conceptual stage	Russo et al. (2026)
Combination therapy strategies	Co-targeting MEOX2 + SRGN	Simultaneously targets proneural GSCs (MEOX2) and mesenchymal GSCs (SRGN)	Eliminates heterogeneous GSC populations, improves GBM efficacy	*In vitro* (GSC proliferation, sphere assays) + *in vivo* (intracranial xenograft mice)	Preclinical (*in vivo* proof-of-concept completed)	Lu et al. (2025)
Combination therapy strategies	MEOX2/GLI1 axis inhibitor + EGFR-TKI or platinum-based chemotherapy	Inhibits MEOX2/GLI1 axis, reverses epigenetic EGFR dysregulation	Overcomes resistance to erlotinib, osimertinib, cisplatin in lung cancer	*In vitro* (NSCLC cell lines: proliferation, colony formation, resistance) + *in vivo* (xenograft mice)	Preclinical (cellular and animal model validation completed)	Armas-López et al. (2017), Peralta-Arrieta et al. (2022)
Indirect intervention (miRNA)	miR-301a mimic (targets oncogenic MEOX2); miR-130a inhibitor (upregulates tumour-suppressive MEOX2)	Modulates MEOX2 expression	Inhibits tumour progression in preclinical models (breast, HCC, etc.)	*In vitro* (cell proliferation, migration, apoptosis assays)	Preclinical	Zhou et al. (2012a), Tang et al. (2025)
Indirect intervention (ceRNA network)	Targeting MEOX2-AS1/miR-143-3p axis	Intervenes in competitive endogenous RNA network	Therapeutic strategy for cervical cancer	*In vitro* (CC cell proliferation, migration, invasion) + *in vivo* (xenograft mice)	Preclinical	Liu et al. (2021a)
Indirect intervention (upstream regulators)	Activate RNF10 or intervene in TGF-β signalling	Indirectly upregulates MEOX2 to exert protective effects	Protective effects in cardiovascular disease (restenosis, cardiotoxicity) and liver fibrosis	*In vitro* (cardiac fibroblasts, H9C2) + *in vivo* (rat restenosis model, THP-induced cardiotoxicity model)	Preclinical	Wang et al. (2024), Li et al. (2019)
Indirect intervention (downstream pathways)	Inhibit PI3K/AKT, ERK, Hh pathways	Alternative treatment option for MEOX2-high tumours	Treatment of MEOX2-overexpressing tumours	*In vitro* (multiple tumour cell lines) + *in vivo* (xenograft mice)	Clinically available drugs (but biomarker-guided strategy remains preclinical)	Schönrock et al. (2022), Peralta-Arrieta et al. (2022), Cai et al. (2022)
Gene therapy	Restoring MEOX2 function in brain endothelial cells (gene replacement)	Restores MEOX2 expression	Improves blood–brain barrier function and Aβ clearance in AD	*In vitro* (human brain microvascular endothelial cells) + *in vivo* (Meox2-KO mice)	Preclinical (proof-of-concept in animal models)	Wu et al. (2005)
Gene therapy	Meox2-Cre-mediated interneuron-specific SCN1A gene replacement (dual-AAV)	Corrects SCN1A deficiency in GABAergic interneurons in DS	Corrects epileptic phenotypes, prevents postnatal death and seizures in mouse models	*In vivo* (conditional Scn1a-KO mice, Scn1a-mutant DS models)	Preclinical (phenotypic correction achieved in mice; not yet in clinical trials)	Mich et al. (2025), Williams et al. (2019)
Cell therapy	Hypoxia-preconditioned human umbilical vein endothelial cell (HUVEC) therapy	Hypoxia upregulates MEOX2 and other genes, enhancing migration and neurovascular protection	Novel cell therapy for neonatal hypoxic-ischaemic encephalopathy	*In vitro* (HUVEC migration, angiogenesis) + *in vivo* (neonatal rat hypoxic-ischaemia model)	Preclinical (rat model validation)	Lee et al. (2018)

## 6 Conclusions and perspectives

MEOX2, an ancient and multifunctional homeobox transcription factor, exemplifies the translational path from developmental biology to disease medicine. This Review presents MEOX2’s physiological functions and its dual-faceted roles in neoplastic and non-neoplastic diseases. In oncology, whether MEOX2 acts as an ‘oncogene’ or ‘tumour suppressor’ is highly dependent on tissue origin and cellular context. In neurodegenerative, cardiovascular, fibrotic and developmental disorders, MEOX2 dysfunction is frequently a key link in disease pathogenesis.

We recognize that the quality and strength of studies addressing MEOX2 vary considerably across different disease contexts. We stratified the evidence into four levels: Level I (large-scale prospective cohorts or RCTs), Level II (retrospective cohort studies with independent validation), Level III (small series or bioinformatics-derived models without external validation) and Level IV (preclinical studies). The strongest evidence for MEOX2 as a prognostic biomarker resides in gliomas, breast cancer and hepatocellular carcinoma, where multiple retrospective cohort studies have consistently demonstrated independent prognostic value (Level II) (Schönrock et al., 2022; Tachon et al., 2019; Wang et al., 2022a; Cai et al., 2022; Zhou et al., 2012b). By contrast, most multi-gene signatures incorporating MEOX2 have been derived solely from bioinformatics analyses without prospective validation, placing them at Level III (Wu et al., 2025; Wang Z. et al., 2025; Zhou et al., 2025; Liu and Liu, 2024; Feng et al., 2024). Therapeutic intervention strategies—including small-molecule inhibitors (Lu et al., 2025), miRNA-based modulation (Zhou et al., 2012a; Tang et al., 2025), gene replacement (Wu et al., 2005; Mich et al., 2025) and cell therapy (Lee et al., 2018)—all remain at the preclinical stage (Level IV). No randomised controlled trials evaluating MEOX2-directed therapies have been reported, representing a major gap that must be addressed in future research.

Despite significant progress, several major challenges remain. First, the mechanistic basis of MEOX2’s functional duality demands deeper analysis beyond the simple assertion of context dependence. This duality can be parsed into two distinct but interacting layers: pathway dependency and tissue dependency. Pathway dependency refers to the specific signalling cascades engaged by MEOX2 in a given cellular state—in GBM and lung cancer, MEOX2 potentiates ERK/GLI1 signalling (Schönrock et al., 2022; Armas-López et al., 2017; Peralta-Arrieta et al., 2022); in breast and laryngeal cancers, it inhibits PI3K/AKT pathways (Cai et al., 2022; Tian et al., 2018). Tissue dependency reflects the pre-existing transcriptional programmes established during development, shaped by tissue-specific co-factor networks (Lin et al., 2005; Valcourt et al., 2007) and epigenetic landscapes (Ávila-Moren et al., 2014; Cortese et al., 2008). These two layers converge to determine net functional outcome, and neither alone is sufficient to predict MEOX2’s role in a given tumour type. Second, targeting specificity and tool scarcity remain formidable obstacles. Distinguishing MEOX2 from its paralogue MEOX1—which shares substantial homeodomain homology (Grigoriou et al., 1995; Schönrock et al., 2022) and partial functional redundancy during development (Reijntjes et al., 2007)—is a major challenge, and no MEOX2-selective inhibitors have been reported. Indirect strategies (miRNAs, RNF10, TGF-β modulation) carry off-target risks, as these networks are shared with normal physiological functions (Chen and Gorski, 2008; Dhahri et al., 2020). Third, practical translational barriers include specimen accessibility, lack of standardised detection techniques—as exemplified by the absence of unified IHC scoring criteria for MEOX2 positivity across studies (Soukup et al., 2023)—and economic considerations for routine implementation. Fourth, MEOX2’s relationship with the tumour microenvironment and inflammatory cells has emerged as an important dimension: it correlates with macrophage infiltration in oesophageal cancer (Wang et al., 2021), HEV-associated T/B-cell infiltration in breast cancer (Sawada et al., 2022), and neutrophil infiltration in rheumatoid arthritis (Yu et al., 2021; Comertpay and Gov, 2020). In GBM, MEOX2 expression correlates with galectin-1 and CD4^+^ T-cell/macrophage patterns (Martínez-Bosch et al., 2023). These observations suggest that MEOX2’s oncogenic versus tumour-suppressive effects may be partially mediated through immune regulation, though the precise mechanisms remain poorly defined.

The challenges discussed above naturally point to several priorities for future investigation. Mechanistically, multi-omics approaches—single-cell multi-omics, spatial transcriptomics, Hi-C and CRISPR screening—could delineate MEOX2’s genome-wide binding landscape and resolve its functional duality across cellular contexts. Translational efforts should focus on two complementary avenues: first, developing selective agents that target MEOX2 protein–protein interaction interfaces (e.g., with RNF10 or SMADs) or use PROTACs and molecular glues to overcome the current lack of MEOX2-specific inhibitors and the challenge of distinguishing it from MEOX1; and second, establishing robust clinical validation frameworks, including large prospective cohorts, standardised detection protocols and exploration of MEOX2 as a liquid biopsy marker, to address the practical barriers currently impeding clinical implementation. Finally, the emerging roles of MEOX2 in the tumour microenvironment and systemic inflammation warrant deeper investigation, as its immune-related functions—correlating with macrophage infiltration, T/B-cell recruitment and neutrophil-associated inflammation—remain mechanistically undefined and could reveal new combination strategies for immunotherapy. Together, these priorities aim to translate the current correlative and preclinical evidence into mechanistic understanding, therapeutic innovation and clinical utility.

In conclusion, MEOX2 is a hub molecule connecting developmental biology to a wide range of human diseases. In-depth study of MEOX2 will deepen our understanding of fundamental life processes and bring new diagnostic tools and therapeutic hope for diseases currently lacking effective treatments (e.g., high-grade glioma, AD, organ fibrosis). With continuous advances in research tools and concepts, MEOX2 is poised to occupy a significant place in the future of precision medicine.

## Glossary

AAV: adeno-associated virus
AD: Alzheimer disease
AML: acute myeloid leukaemia
ASD: autism spectrum disorder
ASO: antisense oligonucleotide
BC: breast cancer
CAF: cancer-associated fibroblast
CC: cervical cancer
ceRNA: competitive endogenous RNA
CGGA: Chinese Glioma Genome Atlas
CHD: coronary heart disease
circRNA: circular RNA
CRC: colorectal cancer
CS: congenital scoliosis
CSF-1: colony-stimulating factor 1
CTSS: cathepsin S
DHEC: dihydroergocryptine
DS: Dravet syndrome
EC: endothelial cell
EGFR: epidermal growth factor receptor
EGFR-TKI: epidermal growth factor receptor-tyrosine kinase inhibitor
EMT: epithelial–mesenchymal transition
ERK: extracellular signal-regulated kinase
ESCC: oesophageal squamous cell carcinoma
Gal-1: galectin-1
GBM: glioblastoma
GC: gastric cancer
GDM: gestational diabetes mellitus
GEO: Gene Expression Omnibus
GSC: glioblastoma stem-like cell
GWAS: genome-wide association study
HCC: hepatocellular carcinoma
HD: Huntington disease
HEV: high endothelial venule
HF: heart failure
HGPS: Hutchinson–Gilford progeria syndrome
Hh: Hedgehog
HUVEC: human umbilical vein endothelial cell
IHC: immunohistochemistry
IPMN: intraductal papillary mucinous neoplasm
KIRC: kidney renal clear cell carcinoma
LC: laryngeal cancer
LF: liver fibrosis
LGG: low-grade glioma
lncRNA: long non-coding RNA
**m** ^**6**^ **A**: N^6^-methyladenosine
miRNA: microRNA
NSCLC: non-small cell lung cancer
NTD: neural tube defect
OS: overall survival
OV: ovarian cancer
PH: pulmonary hypertension
PI3K: phosphatidylinositol 3-kinase
PROTAC: PROteolysis TArgeting Chimera
qPCR: quantitative real-time polymerase chain reaction
RA: rheumatoid arthritis
RNF10: RING finger protein 10
SNP: single-nucleotide polymorphism
TCGA: The Cancer Genome Atlas
TGF-β: transforming growth factor-beta
TME: tumour microenvironment
TNBC: triple-negative breast cancer
VSMC: vascular smooth muscle cell
